# Recent developments in catalysis and inhibition of the Jumonji histone demethylases

**DOI:** 10.1016/j.sbi.2023.102707

**Published:** 2023-10-11

**Authors:** Letitia Sarah, Danica Galonić Fujimori

**Affiliations:** 1 Chemistry and Chemical Biology Graduate Program, University of California San Francisco; San Francisco, CA 94158, USA; 2 Department of Cellular and Molecular Pharmacology, University of California San Francisco; San Francisco, CA 94158, USA; 3 Department of Pharmaceutical Chemistry, University of California San Francisco; San Francisco, CA 94158, USA; 4 Quantitative Biosciences Institute (QBI), University of California San Francisco; San Francisco, CA 94158, USA

**Keywords:** Jumonji histone demethylases, Domain crosstalk, Catalysis, Post-translational modifications, Lysine demethylation, Orthosteric inhibitors, Allosteric inhibitors

## Abstract

Histone methylation, one of the most common histone modifications, has fundamental roles in regulating chromatin-based processes. Jumonji histone lysine demethylases (JMJC KDMs) influence regulation of gene transcription through both their demethylation and chromatin scaffolding functions. It has recently been demonstrated that dysregulation of JMJC KDMs contributes to pathogenesis and progression of several diseases, including cancer. These observations have led to an increased interest in modulation of enzymes that regulate lysine methylation. Here, we highlight recent progress in understanding catalysis of JMJC KDMs. Specifically, we focus on recent research advances on elucidation of JMJC KDM substrate recognition and interactomes. We also highlight recently reported JMJC KDM inhibitors and describe their therapeutic potentials and challenges. Finally, we discuss alternative strategies to target these enzymes, which rely on targeting JMJC KDMs accessory domains as well as utilization of the targeted protein degradation strategy.

## Introduction

Chromatin is a complex of histone proteins and DNA that allows condensed packaging of the genetic material in eukaryotic cells [1]. The accessibility of chromatin regulates gene transcription and is mediated via an interplay of various transcriptional regulators, including chromatin remodelers and enzymes that catalyze post-translational modifications of chromatin. Among these modifications, methylation of histone lysine residues has a profound effect on chromatin accessibility [2]. Methylation of lysines is dynamically regulated by the interplay between histone methyltransferases (KMTs, methylation writers) and histone demethylases (KDMs, methylation erasers). Two families of KDMs have been identified and characterized based on their catalytic mechanisms: the flavin-dependent lysine demethylases (LSDs or KDM1s) and the Fe(II)-dependent Jumonji C-domain containing lysine demethylases (JMJC KDMs) [3]. Deregulation of these modifiers is one of the most common alterations in human cancers and influences key processes in oncogenesis encompassing cell growth, immune evasion, metastasis, transcriptomic heterogeneity, and drug resistance [4]. An in-depth understanding of the catalytic and non-catalytic functions of these proteins is required to understand their functions, both in the normal cell and disease setting. Targeting histone demethylases is increasingly being recognized as an attractive therapeutic strategy for cancer as well as neurological, autoimmune, and cardiovascular diseases [5]. Unlike small-molecule modulators of KMTs and LSD1s, which in some cases have advanced to the clinic, JMJC KDMs modulators are yet to be approved for disease treatment [6,7]. In this review, we discuss recent progress on understanding regulation of JMJC KDMs catalysis and development of small molecule inhibitors targeting both catalytic and auxiliary domains of these enzymes. For a more comprehensive discussion of JMJC KDMs catalysis, inhibition, and cellular function, we refer readers to several in-depth reviews [1,8-11].

## Recent development in KDM catalysis

### Paralog-specific acidic patch recognition by KDM2 family demethylases

**Figure 1:**
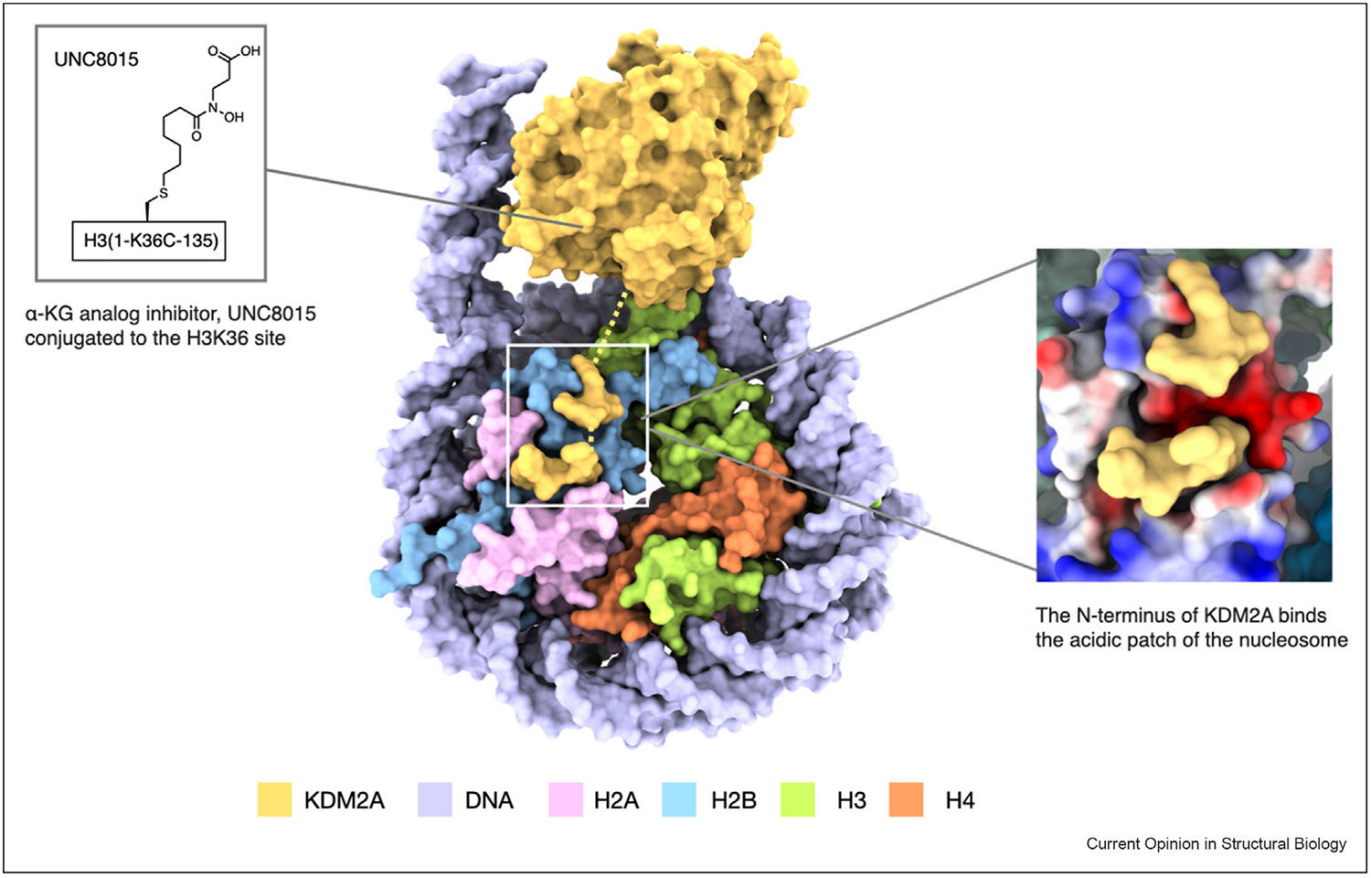
Cryo-EM structure of KDM2A interacting with the acidic patch of the nucleosome (PDB 7UV9). Conjugation of the α-KG analog inhibitor (UNC8015) to the H3K36 site revealed the paralog-specific recognition of the acidic patch of the nucleosome by the N-terminus of KDM2A but not KDM2B.

The JMJC domain-containing demethylase family comprises of about 20 human enzymes that are classified into KDM2-8 families [5,12,13]. Members can catalyze the removal of methyl groups from mono-, di- and trimethylated lysines, using α-ketoglutarate and oxygen as co-substrates and Fe(II) as co-factor. While the enzymes in this family share a largely conserved iron-containing active site harbored within the JmjC domain, they differ in auxiliary domains, which typically interact with DNA or histone proteins. Although KDMs have been studied extensively in vitro using peptide substrates, their ability to interact with and demethylate nucleosomes is relatively poorly understood. Structures of KDMs in complex with nucleosomes can provide insights into the substrate recognition beyond the histone tails [14]. A recent report has characterized a paralog-specific mechanism of nucleosome interaction for enzymes in the KDM2 subfamily [15]. Cryo-EM structures of KDM2A and KDM2B with nucleosomes modified to incorporate an α-ketoglutarate mimic in place of methylated Lys36 in histone H3 has revealed recognition of the nucleosome’s acidic patch by the N-terminus of KDM2A but not KDM2B (Figure 1). This study exemplifies the need to investigate the enzymatic mechanism of the histone-modifying proteins on the nucleosome level rather than on the histone peptides to capture the dynamic and multivalent interactions between enzymes and chromatin.

### XLID mutations disrupt chromatin sensing by KDM5C

**Figure 2:**
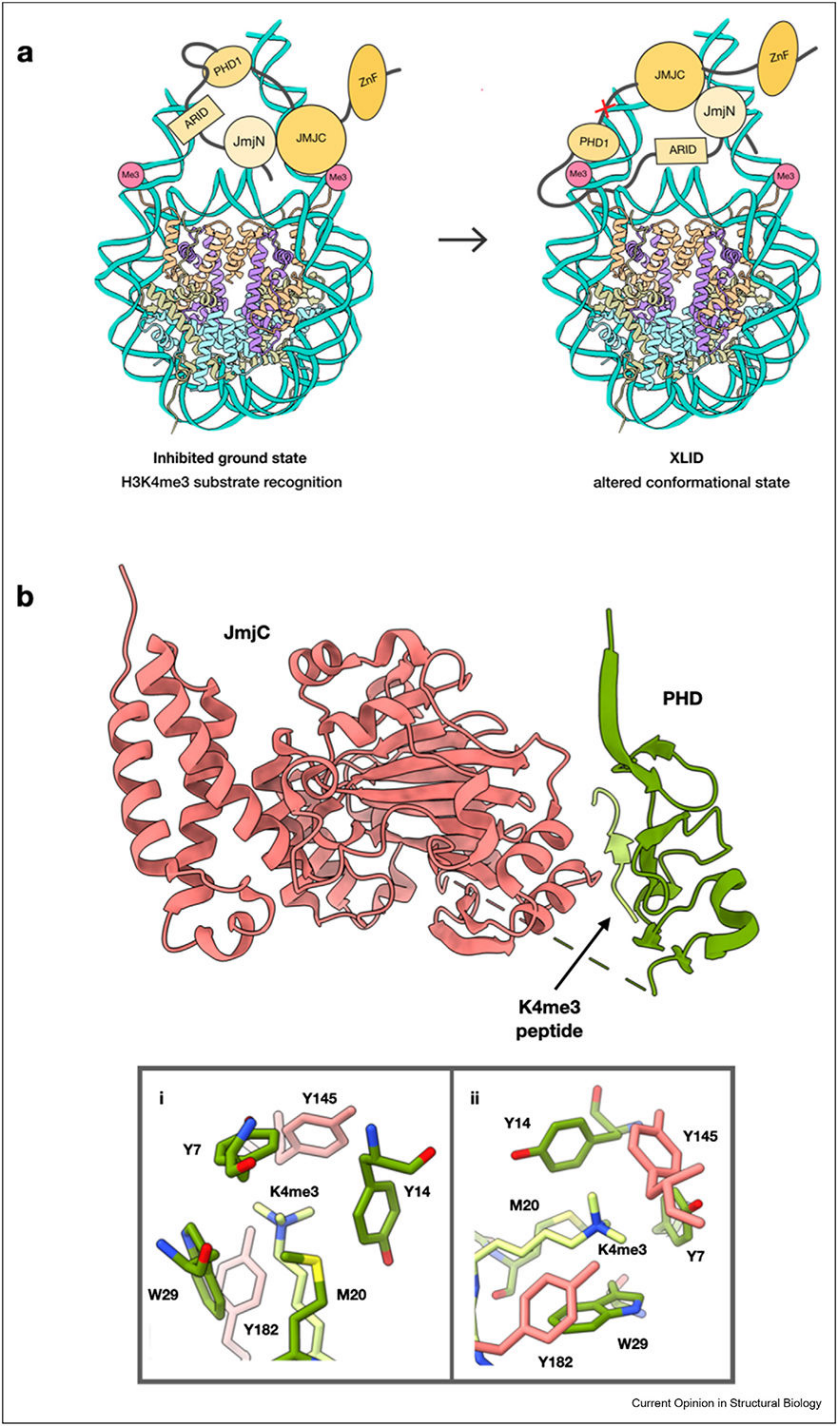
Regulation of chromatin recognition and demethylation in KDM5C and PHF2. **a**, XLID mutations (X) influence altered conformational state of the ARID and PHD1 region in KDM5C leading to an increase of non-productive chromatin engagement and dysregulation of demethylation. **b**, N-terminal

The *KDM5C* gene, which regulates neuronal genes expression, features several mutations adjacent to its ARID and PHD1 domains that cause X-linked intellectual disability (XLID) [16-19]. Recent findings show that the unstructured linker region between these two domains is necessary for nucleosome binding, and that its accessibility is regulated by PHD1. XLID mutations adjacent to the ARID and PHD1 domains disrupt this regulation, such that nucleosome binding is enhanced but specificity towards H3K4me3 is lost (Figure 2a). This work highlights mechanistic differences between the members of the KDM5 family. Unlike the PHD1 domain of KDM5A which allosterically stimulates catalysis by binding the product of demethylation, the PHD1 of KDM5C has an inhibitory role in catalysis [20,21]. Additionally, while the ligand preference of PHD1 domains for unmodified H3 tail is conserved across studied members of the family, the affinity is substantially different, with PHD1 domains of KDM5A and KDM5B showing sub-micromolar binding affinity, which is over 100-fold reduced in the PHD1 domain of KDM5C [22-24].

### H3P16oh enhanced KDM5A chromatin recruitment and demethylation

Recruitment of demethylases to chromatin regulates the ability of these enzymes to carry out demethylation. A recently reported investigation led to evidence for hydroxylation of proline at residue 16 of histone H3 in mammalian cells catalyzed by the hypoxia inducible factor (HIF) prolyl hydroxylase EGLN2 [25]. Although further validation is desirable due to discrepancies in reports on EGLN substrates [26], this observation is interesting because of the validated roles of EGLN prolyl hydroxylases in regulation of the hypoxic response and of growing evidence for oxygen availability mediated regulation of chromatin methylation status [27-30]. This hydroxylated proline modification enhances direct binding of KDM5A to its substrates, H3K4me3 modified nucleosomes. The enhanced recruitment to chromatin results in a decrease in H3K4me3 at the promoter of target genes.

### H3K4me3 recognition and its effect on H3K9me2 demethylation by PHF2

PHF2 is a member of the KDM7 family which binds to H3K4me3 through its PHD domain and demethylates H3K9me2/me1 via its Jumonji domain [31-33]. A recent study describes how interaction of PHF2 with H3K4me3 influences its demethylase activity [34]. In PHF2, residues from both the PHD and the Jumonji domain contribute to H3K4me3 binding interface (Figure 2b). The N-terminal segment of PHF2 harboring both domain binds to H3K4me3 peptide with a K_D_ of 160 nM, an affinity 4-fold higher than that of the isolated PHD domain of this enzyme. This finding demonstrates that additional domains of chromatin modifying enzyme may be necessary for optimal engagement of histone tail ligands by the PHD reader domains.

### KDM5 interactomes and non-histone substrates

In addition to regulating transcription through their histone demethylase activity, JMJC KDMs also have catalysis-independent gene regulatory functions that remain less characterized. These functions are often related to JMJC KDMs being assembled into multi-subunit complexes to enable coordinated action of distinct activities necessary for chromatin regulation [35]. In pursuit of identifying the interacting partners of KDM5A, several groups used co-immunoprecipitation and mass spectrometry techniques and reported stable association of KDM5A with the SIN3B-EMSY and Nucleosome Remodeling and Deacetylase (NuRD) complexes in mammalian cells [35,36]. Additionally, another group utilized Turbo-ID mediated proximity labeling in *Drosophila melanogaster* to capture the interactomes of the Drosophila KDM5 homolog, Lid [37]. The enzyme was found to interact with members of the switch/sucrose non-fermentable (SWI/SNF) and nucleosome remodeling factor (NURF) complex, the non-specific lethal (NSL) complex, mediator, and several insulator proteins, suggesting a complex transcriptional regulatory network that Lid contributes to. Taken together these findings support the idea that the interaction between KDM5A and NuRD is conserved across multiple organisms. Complementary to the effort of identifying KDM5 interacting partners, JMJC KDMs have also been reported to have non-histone substrates [38,39]. Although the relevance of KDM5 activity to disease progression has been primarily established through its ability to regulate gene expression via histone methylation, these enzymes may also target non-histone proteins, as demonstrated by in vitro activity towards a library of 180 permutated peptide substrates derived from the H3K4me3 sequence [39]. Of these substrates, p53-K370me3 is particularly notable as a putative non-histone substrate. Further studies are needed to establish functional relevance of this modification.

### Recent developments in Jumonji domain inhibitors

**Figure 3:**
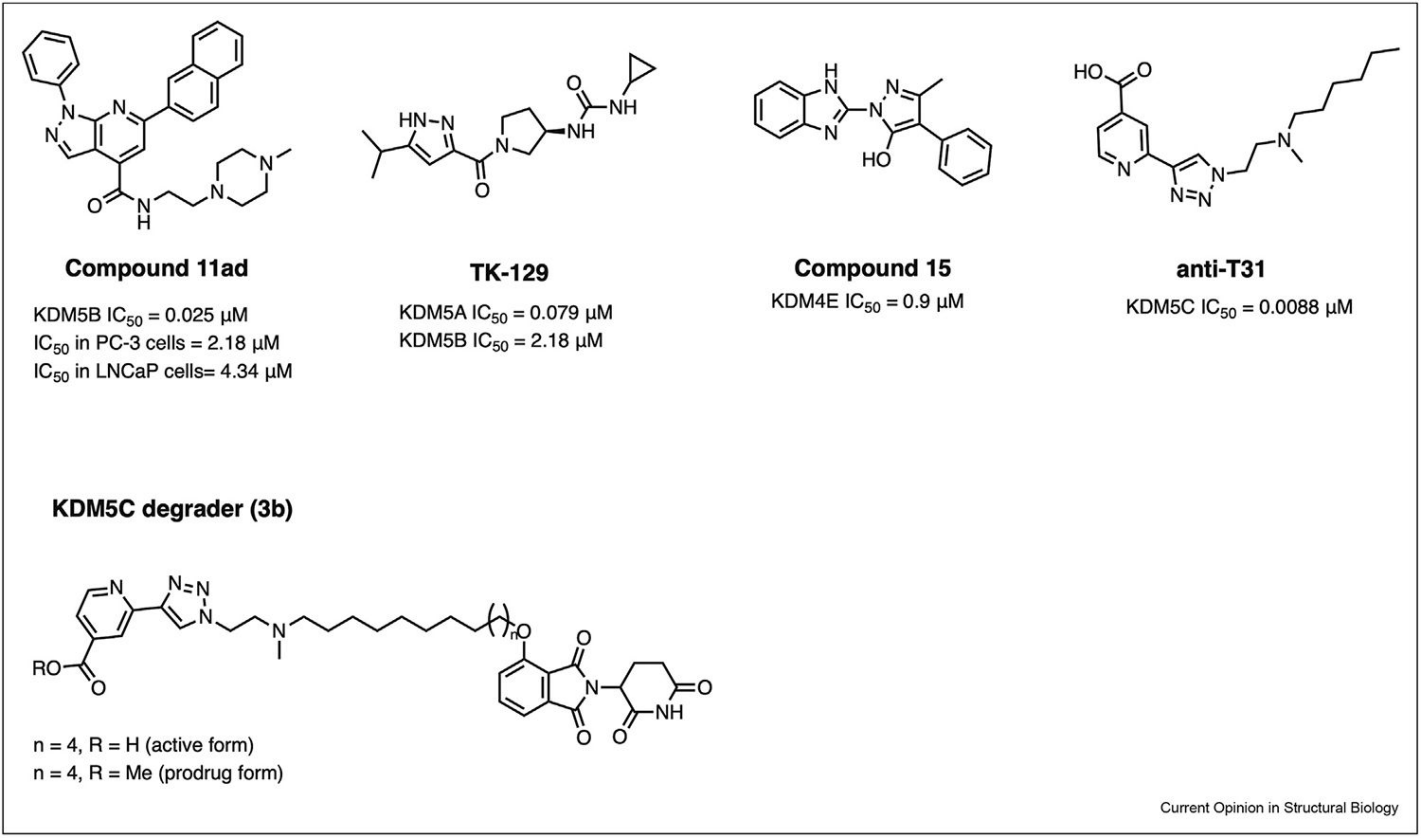
Examples of recently developed inhibitors and degraders targeting Jumonji histone demethylases. Sources for IC50 values are cited in the main text.

Development of JMJC KDM inhibitors has been a focus of several recent studies. These efforts are supported by progress in solving JMJC KDM structures, especially through crystallography. Most of the recently reported small molecule inhibitors target the active site of JMJC KDMs and coordinate the active site Fe(II). The proposed mechanism of action for most of these active site inhibitors is via competition with α-ketoglutarate, an essential co-substrate of JMJC KDMs [1,40,41]. One compound in this class of inhibitors is GS-5801, which failed in an early phase clinical trial due to tolerability concerns [42]. Moreover, a pan-KDM4 inhibitor, TACH101 is currently in phase 1 clinical trial for patients with advanced and metastatic tumors [43]. As of now GS-5801 and TACH101 remain the only two JMJC KDM inhibitors to have reached the clinic and this further underscores the need for developing JMJC KDM inhibitors. Encouragingly, the development of small molecule inhibitors of enzymes in this class continues to be an active effort, with the most recent developments described here (Figure 3).

Of the more canonical active site inhibitors, Tang et al. have recently described the development of a novel and highly potent pyrazole-based KDM5B inhibitor **TK-129** [44]. The inhibitor was shown to have protective effects on myocardial remodeling and fibrosis through the inhibition of KDM5B and the downstream KDM5B-regulated Wnt pathway. Furthermore, another recent publication showed a novel 1H-pyrazole-[3,4-b] pyridine-based KDM5B inhibitor [45]. The best performing analog of the series, **11ad** inhibits KDM5B in vitro and in vivo, and demonstrates activity against prostate cancer via inhibition of the PI3K/AKT pathway.

Utilizing an azide—alkyne cycloaddition reaction, Miyake et al. report a novel KDM5C inhibitor. This reaction leverages Fe(II) as a catalyst, a novel strategy for in situ formation of demethylase inhibitors [46]. The resulting triazole-based inhibitor, **anti-T31**, was developed following a high-throughput activity-based screen of a combinatorial fragment library of mixed alkynes and azides. The methyl ester prodrug analog of the inhibitor, **anti-T31’** showed activity in cells by inhibiting KDM5C and inducing higher levels of H3K4me3. Future studies are needed to evaluate if the in-situ inhibitor formation strategy used in this approach is generalizable to other Jumonji demethylases.

With the objective of developing inhibitors of KDM4 family, Carter and colleagues detail the development of a benzimidazole benzylpyrazole class of inhibitors [47]. Unexpectedly, a representative inhibitor, **compound 15**, does not compete with either α-ketoglutarate or the peptide substrate. Instead, compound 15 primarily exerts its inhibitory activity by competing with the enzyme for binding of Fe(II), consistent with its bidentate chelator features. Additionally, structural studies have revealed that the inhibitor engages a distal site on the enzyme surface, which is structurally conserved amongst all members of the KDM4 family. While the functional relevance of this site is yet to be established, the authors hypothesize that binding of the inhibitor to this site could restrict enzyme dynamics to prevent substrate turnover.

While inhibiting JMJC KDM catalytic site is a promising strategy for drug discovery, only a subset of the functional roles of JMJC KDMs in cancer are mediated through their catalytic activity. Emerging evidence suggests that in some instances, oncogenic transcription is promoted by the ability of JMJC KDMs to scaffold transcriptional regulators to chromatin. For example, demethylase KDM5B acts in a catalysis-independent manner to recruit methyltransferase SETDB1 to inhibit expression of retroelements and type I interferon response genes, leading to suppression of anti-tumor immunity [48]. These examples emphasize the need for development of demethylase-targeting modalities that abrogate their scaffolding functions. Recently, proteolysis-targeting chimeras (PROTACs) have emerged as a pharmacological approach to abrogate all functionalities of proteins, including catalytic and scaffolding function [49]. Encouragingly, small molecule degraders for KDM5C have been reported recently [50]. The reported compound, **3b**, induced degradation of KDM5C through the ubiquitin proteasome pathway. This is the first JMJC KDM degrader reported, and it provides insights into designing degraders for other JMJC KDM family proteins.

### Targeting demethylases beyond their catalytic domains

**Figure 4:**
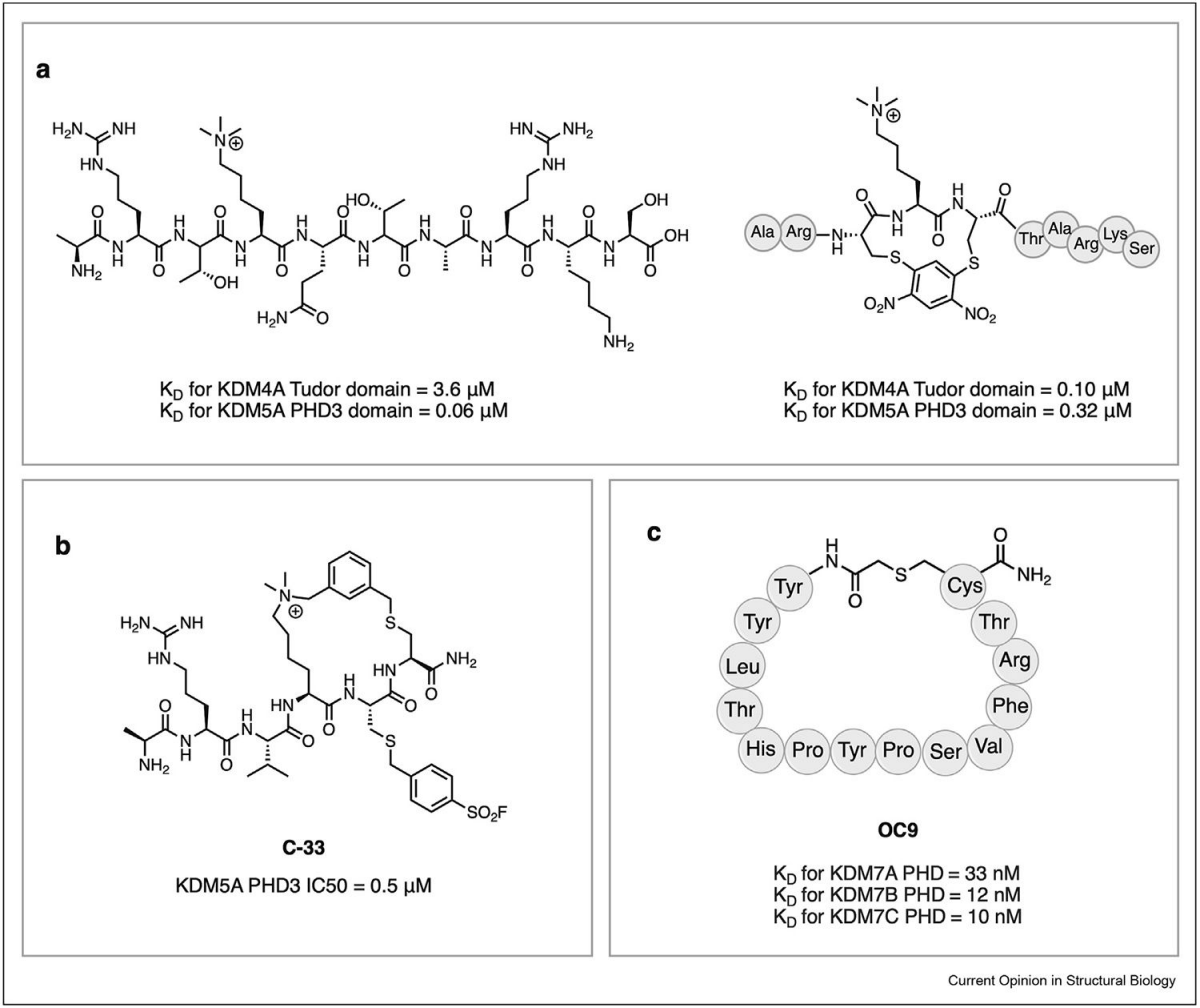
Cyclic peptides targeting non-catalytic domains of JMJC KDMs. **a,** Stapled peptide ligands for PHD3 domain of KDM5A and Tudor domain of KDM4A. **b,** Proximity-reactive covalent ligand for PHD3 domain of KDM5A. **c,** Structure of OC9, a cyclic peptide targeting PHD finger of KDM7 subfamily.

In addition to the catalytic domain, Jumonji demethylases contain multiple additional domains, including Tudor domain, F-box domain, plant homeodomain (PHD), zinc finger, AT-rich interaction domain (ARID) and leucine-rich repeat (LRR) domains [51]. Amongst the various domains, inhibitor discovery efforts have mostly been directed towards targeting the chromatin reader domains, including Tudor domains and PHD domains. This is partly due to the structural information enabled by the X-ray and NMR structures available for these domains [22,52,53]. Additionally, divergent domain architecture across subfamilies of demethylases makes auxiliary domains appealing targets for selective chemical probes. Betlem et al. recently reported the development of stapled histone trimethyl lysine peptides for the Tudor domain of KDM4A and the PHD3 domain of KDM5A, both of which recognize trimethylated Lys4 of histone H3 (H3K4me3) (Figure 4a) [54]. When compared to the binding affinity of the corresponding linear peptides, the stapled peptides display increased binding affinity for Tudor domain of KDM4A and decreased affinity for PHD3 domain of KDM5A, suggesting that constrained histone peptides have higher level of selectivity compared to linear peptides.

Zhang et al. recently reported the development of covalent cyclic peptide inhibitors to target exposed lysine residues adjacent to the H3K4me3 binding site in the PHD3 domain of KDM5A [55]. The most potent analog of the cyclic peptide series, **C33**, bears an aryl sulfonyl fluoride and displays sub-micromolar binding to the PHD3 domain (Figure 4b). Furthermore, this cyclic peptide inhibitor displays >500-fold selectivity over the PHD1 finger of KDM5A, which binds unmodified H3K4. Further optimization is required to enhance cell permeability of these peptides.

More recently, Coleman et al. described the discovery of **OC9**, a cyclic peptide targeting the PHD domains of KDM7 family (Figure 4c) [56]. **OC9** is described to disrupt the PHD-H3K4me3 interaction by engaging the aromatic cage of the KDM7’s PHD domains through a valine residue in the cyclic peptide. PHD-finger inhibition by **OC9** differently regulates demethylation of H3K9me2 by KDM7 enzymes. OC9 has opposing effects on the catalytic activity of KDM7 demethylases. While the presence of this cyclic peptide inhibits KDM7B-catalyzed demethylation of Lys9 in H3K4me3K9me2 peptide, KDM7A-catalyzed demethylation of the same substrate is strongly stimulated by the cyclic peptide. These results underscore the potential of targeting PHD domain of JMJC KDMs to selectively modulate of demethylase activity via allosteric regulation.

## Conclusions and future directions

JMJC histone demethylases often contain multiple domains which allows for regulation of demethylation through recognition of chromatin environment, enabled by the cross talk between the catalytic and auxiliary domains [57]. Utilizing multi-domain constructs to investigate regulation of JMJC KDMs will better recapitulate the complex mechanisms that control chromatin methylation. Further work is needed to advance pharmacological tools that perturb demethylase function, both through targeting catalytic and allosteric domains. This latter approach is likely to improve selectivity, a challenge often faced by orthosteric ligands. While recent studies have established cyclic peptides as well-suited modality for targeting of the reader domains embedded within demethylases, additional efforts are required to develop small molecule modulators of these extended, highly solvent accessible chromatin interacting modules. Finally, differential surface presentation of exposed Lys residues offers a potential for paralog-selective degraders, a concept that awaits experimental validation.

## Data Availability

No data was used for the research described in the article.
